# Long-term monitoring reveals carbon–nitrogen metabolism key to microcystin production in eutrophic lakes

**DOI:** 10.3389/fmicb.2015.00456

**Published:** 2015-05-12

**Authors:** Lucas J. Beversdorf, Todd R. Miller, Katherine D. McMahon

**Affiliations:** ^1^Department of Civil and Environmental Engineering, University of Wisconsin-MadisonMadison, WI, USA; ^2^Joseph J. Zilber School of Public Health, University of Wisconsin-MilwaukeeMilwaukee, WI, USA; ^3^Department of Bacteriology, University of Wisconsin-MadisonMadison, WI, USA

**Keywords:** microcystin, *Microcystis*, NtcA, 2-oxoglutarate, carbon, nitrogen

## Abstract

The environmental drivers contributing to cyanobacterial dominance in aquatic systems have been extensively studied. However, understanding of toxic vs. non-toxic cyanobacterial population dynamics and the mechanisms regulating cyanotoxin production remain elusive, both physiologically and ecologically. One reason is the disconnect between laboratory and field-based studies. Here, we combined 3 years of temporal data, including microcystin (MC) concentrations, 16 years of long-term ecological research, and 10 years of molecular data to investigate the potential factors leading to the selection of toxic *Microcystis* and MC production. Our analysis revealed that nitrogen (N) speciation and inorganic carbon (C) availability might be important drivers of *Microcystis* population dynamics and that an imbalance in cellular C: N ratios may trigger MC production. More specifically, precipitous declines in ammonium concentrations lead to a transitional period of N stress, even in the presence of high nitrate concentrations, that we call the “toxic phase.” Following the toxic phase, temperature and cyanobacterial abundance remained elevated but MC concentrations drastically declined. Increases in ammonium due to lake turnover may have led to down regulation of MC synthesis or a shift in the community from toxic to non-toxic species. While total phosphorus (P) to total N ratios were relatively low over the time-series, MC concentrations were highest when total N to total P ratios were also highest. Similarly, high C: N ratios were also strongly correlated to the toxic phase. We propose a metabolic model that corroborates molecular studies and reflects our ecological observations that C and N metabolism may regulate MC production physiologically and ecologically. In particular, we hypothesize that an imbalance between 2-oxoglutarate and ammonium in the cell regulates MC synthesis in the environment.

## Introduction

Global climate change is expected to increase the occurrence and risk of human exposure to environmental pollutants and toxins, including those associated with cyanobacterial harmful algal blooms (cyanoHABs) (Paerl and Huisman, 2009; Balbus et al., 2013). The success of cyanobacteria thus far has been associated with environmental factors such as elevated water temperature, eutrophication and altered nutrient stoichiometry, and thermal stratification of lakes, as well as physiological adaptations in the ability of cyanobacteria to control their buoyancy, store nutrients, and maximize light use and carbon fixation (Hyenstrand et al., 1998). The factors associated with cyanobacterial dominance are now widely accepted, for the most part. However, very little is known about the functional and/or ecological role of cyanotoxin production, or the variables that contribute to increased toxin production in natural systems.

The most cosmopolitan, toxin-producing cyanobacteria belong to the genus *Microcystis*, and the microcystins (MC) are ubiquitous liver toxins in eutrophic lakes that threaten water quality worldwide. Ecosystem-based studies have implicated some of the key factors involved in the proliferation of *Microcystis* blooms, including nitrogen (N), phosphorus (P), light, pH, water temperature, and N: P ratios (Wicks and Thiel, 1990; Kotak et al., 2000; Oh et al., 2001). However, complex community interactions make it difficult to separate the factors that regulate growth from those involved in microcystin synthesis. As a result, several studies have investigated the environmental factors that differentially affect toxic vs. non-toxic populations by amplifying specific microcystin synthetase genes (Rantala et al., 2006; Yoshida et al., 2007; Rinta-Kanto et al., 2009). Still, the presence of a gene does not always translate to toxin production (Beversdorf et al., 2015). For instance, disruption of just one of the *mcy* genes by deletion, recombination, or transformation due to gene disruption or inactivation by transposons or phage would inhibit microcystin synthesis (Kurmayer et al., 2003, 2004; Christiansen et al., 2008). Fewer studies have directly investigated the expression of microcystin gene transcripts in aquatic systems (Gobler et al., 2007; Sipari et al., 2010; Wood et al., 2011), and improved technologies should facilitate more ecosystem-based transcriptional work, including metatranscriptomics.

The physiological role of microcystin still eludes us despite decades of research. A recent review by Holland and Kinnear (2013) discussed the various physio-ecological roles that have been put-forth for cyanotoxins. Among them, microcystins are hypothesized to aid in grazing defense, allelopathy, nutrient uptake (including iron scavenging), protection from reactive oxygen species (ROS), carbon–nitrogen metabolism, cell-signaling, and overall maintenance of cell homeostasis. Holland and Kinnear concluded that a more systematic approach is needed to deduce the factors involved in toxin production, including validating field findings with culture-based studies that describe the transcription of the gene clusters under the same environmental conditions.

Most of what we know about microcystin production has come from laboratory work performed on *Microcystis aeruginosa*, a model toxin-producing cyanobacterium, and much has been learned from molecular studies of this species, including the known biosynthetic pathway for microcystin production (Tillett et al., 2000). A recent review by Neilan et al. (2012) discussed the various environmental factors that may contribute to cyanotoxin production at a molecular level. N, P, light, pH, growth temperature, and trace metals have all recently been shown to affect microcystin levels within *M. aeruginosa*. This suggests regulation of microcystin synthesis is controlled by global transcriptional regulator(s) responding to multiple environmental and/or physiological cues.

The identification of transcriptional regulators of microcystin synthesis and internal signals that modulate their activity is becoming clearer. Recent studies have begun to interrogate the regulatory regions of the *mcy* operon, which contains known transcriptional start sites (Kaebernick et al., 2002), including a binding site for the global N regulator, NtcA (Ginn et al., 2010). Ginn et al. (2010) reported transcriptional activation of *mcyB* and *ntcA* by *M. aeruginosa* under N stressed conditions, and Sevilla et al. (2010) showed that an excess of N, in the form of nitrate, increased growth but did not influence *mcy* transcription or microcystin production. In an earlier batch culture study, Downing et al. (2005) suggested that microcystin production was directly related to nitrate uptake and even went on to speculate on the importance of 2-oxoglutarate (2-OG) and ammonium (NH^+^_4_) as modulators of microcystin production. And then later, Kuniyoshi et al. (2011) demonstrated that 2-OG increased the affinity of NtcA for the *mcyA* promoter region of *M. aeruginosa*. All of these studies could be directly related to the glutamine synthetase/glutamate synthase pathway (GS-GOGAT), which is directly related to the amount of carbon (2-OG) and N balance in the cell (Muro-Pastor et al., 2001; Lindell et al., 2002; Vázquez-Bermúdez et al., 2002). In cyanobacteria, when intracellular NH^+^_4_ drops below a certain threshold, 2-OG accumulates, binds to NtcA and triggers the expression of genes involved in N assimilation (Ohashi et al., 2011), which requires the P_*II*_ protein and PipX (Paz-Yepes et al., 2003). Thus, under N stressed conditions (or excess C), nitrate (and other forms of N) is taken up until enough NH^+^_4_ is generated to restore the C: N balance within the cell. In a comparative study of toxic and non-toxic *Microcystis* strains, both nitrate transport (*nrtA*) and carbon-concentrating genes (*ccmK3* and *ccmL*) were significantly up-regulated in the toxic strains compared to the non-toxic strains, while genes involved in carbon uptake were down-regulated (Alexova et al., 2011b). Similar studies have linked microcystin production to C availability and hypothesized direct links to photosynthesis through cyanobacterial carbon concentrating mechanisms (CCM) (Jähnichen et al., 2007), RubisCO-related protein binding (Zilliges et al., 2011), glycogen storage (Meissner et al., 2015), altered ratios of photosystem I and II (Makower et al., 2015), and localization of microcystin to the thylakoid membrane (Young et al., 2005). In other words, regulation of microcystin production appears to involve both C and N regulation, including intracellular C: N ratios through the uptake of N and sequestering of C.

In a previous study, we reported that inorganic N was a major driver of cyanobacterial population dynamics in the eutrophic lake, Lake Mendota (South Central Wisconsin, USA) and suggested that N stress may stimulate toxic blooms of *Microcystis* (Beversdorf et al., 2013). Here, we aimed to combine 3 years of toxin measurements to define a period of optimum toxin production in this lake. We then used two long-term ecological research (LTER) datasets to elucidate the factors most likely to contribute to microcystin production in Lake Mendota over intra-annual time scales. We hypothesize that the toxic phase of the lake will be defined by a lack of NH^+^_4_, resulting in high C: N ratios due to an imbalance of 2-OG and NH^+^_4_. We then propose a metabolic pathway for how C and N metabolism influence microcystin production and suggest a physio-ecological role for microcystin production in eutrophic lakes.

## Methods

### Study site

Lake Mendota is a eutrophic lake in south-central Wisconsin, USA, which suffers from chronic, noxious cyanobacterial blooms. It is a medium-sized lake (40 km^2^; 25.3 m max depth) and the first of four eutrophic lakes spanning the Yahara River chain within the Yahara Watershed. The watershed itself is mostly agricultural and contributes massive nutrient inputs to the lakes following spring thaw, while the city of Madison, and surrounding metropolitan area, contributes to urban run-off (Lathrop, 2007). As such, Lake Mendota is subject to massive algal blooms, which are dominated by cyanobacterial biomass (>90% of the phytoplankton community) during the summer months (Beversdorf et al., 2013).

Lake Mendota is also one of the most studied lakes in the world (Brock, 1985). It is one of several Wisconsin lakes included in the North Temperate Lakes-Long Term Ecological Research (NTL-LTER) program and beginning in 1995, has undergone regular sampling at the “Deep Hole” location, which includes multiple biological, chemical, and physical characteristics of the water column. In addition to the NTL-LTER program, the LTER Microbial Observatory (MO; years 2000–2010) was established to monitor changes in microbial community dynamics and complement NTL-LTER observations. Additionally, beginning in 2006, a moored buoy has occupied the Deep Hole location during the ice-off season and records high-resolution (minutes–hours) water temperature profiles, dissolved oxygen, chlorophyll, phycocyanin, wind speed, wind direction, and air temperature. Finally, from 2009 to 2011, we established a field campaign to specifically measure, on weekly time-scales, cyanobacterial community dynamics and cyanotoxin concentrations.

### Analytical measurements

All protocols for the 2009–2011 field seasons are described in Beversdorf et al. (2013), with the exception that 2009 samples were collected at discrete depths using a Van Dorn bottle within in the photic zone and then integrated for comparison to the 2010–2011 photic zone samples that were collected with an integrated sampler. All other chemical (including microcystin), biological, and physical measurements were identical from 2009 to 2011. Microcystin (MC) was extracted by lyophilizing frozen, unfiltered water samples. The remaining pellet was resuspended in 5% acetic acid, freeze thawed three times (–20°C and room temperature, respectively), and then separated by solid phase extraction (Bond Elut C18 column, Varian). After washing with 20% methanol, the final product was eluted in 100% methanol, evaporated in a 55°C oven to dryness, and resuspended in 1 mL of 70% methanol (Harada et al., 1988). MC was then detected and quantified by the Wisconsin State Lab of Hygiene (SLOH) using electrospray ionization-tandem mass spectrometry (API 3200, MS/MS) after liquid chromatography (LC) separation on a Phenomenex Luna C18 column (Eaglesham et al., 1999; Hedman et al., 2008). Overall, we targeted four MC variants in this study—MC-LA, MC-LR, MC-RR, and MC-YR—with MC-LR being the predominant variant in all Lake Mendota samples (L = leucine, R = arginine, A = alanine, and Y = tyrosine). Here, we present MC as the sum of those detected microcystin variants.

All NTL-LTER sampling and preservation procedures, analytical protocols, and detection limits are available online (https://lter.limnology.wisc.edu/research/protocols). Briefly, all nutrients were measured spectrophotometrically using autoanalyzers. N and P measurements from 1995 to 2006 were performed using a Technicon segmented flow autoanalyzer; after 2006, measurements were conducted on an Astoria-Pacific Astoria II segmented flow autoanalyzer. All silica (Si) measurements were performed using a Bausch and Lomb spectrophotometer from 1995 to 2006 and then a Technicon Autoanalyzer II after 2006. Similarly, all C measurements from 1995 to 2006 were performed on OI 700 Carbon Analyzer and after 2006, were measured using a Shimadzu TOC-V_*CSH*_ Carbon Analyzer. Briefly, NH^+^_4_ was measured spectrophotometrically at 660 nm after conversion to indophenol. Nitrate and nitrite (N + N) were simultaneously measured spectrophotometrically at 520 nm following cadmium reduction. Dissolved reactive P (DRP) was measured spectrophotometrically at 880 nm following conversion to the phosphomolybdenum complex. Total P (TP) and total N (TN) were first digested by addition of sodium hydroxide-potassium persulfate and then autoclaving; TP and TN were then measured as DRP and N + N, respectively. Dissolved reactive silica (DRSi) was determined using the heteropoly blue method and read spectrophotometrically at 820 nm. Dissolved (DIC) and total inorganic carbon (TIC) were extracted using phosphoric acid, and dissolved (DOC) and total organic carbon (TOC) were digested using sodium persulfate and heat before detection by a non-dispersive infrared detector (NDIR). We report total carbon (TC) as the sum of TIC and TOC. All nutrient ratios are reported as mass: mass per volume ratios.

### Cyanobacterial community composition

Phytoplankton cell counts were collected by the NTL-LTER program and then enumerated by PhycoTech, Inc., following the 2-hydroxypropyl methacrylate (HPMA) method (Crumpton, 1987). Briefly, 0–8 m integrated lake water samples were collected and preserved in glutaraldehyde (2.5–5.0% final concentration). Samples were filtered onto 0.45 μm nitrocellulose membrane filters, which were then heat-fixed onto glass slides with the acrylate resin. Cell counts were then enumerated using an Olympus BX60, research-grade compound microscope equipped with Nomarski and phase optics, a 1.25–2× multiplier, epifluorescence (blue, green, and UV excitation), and a trinocular head mounted with an Olympus MicroFire™ Digital Camera. PhycoTech, Inc. also converts cell counts to biovolume concentrations (μm^3^ mL^−1^) by multiplying cell density (cells mL^−1^) by the average cell volume (μm^3^ cell^−1^).

Cyanobacterial community composition was also determined using the cyanobacterial phycocyanin intergenic spacer (PC-IGS) region described in Beversdorf et al. (2013). Briefly, this cyanobacterial-specific analysis exploits the variable PC-IGS region of the phycocyanin operon. Following *MspI* digestion, the variable lengths of the PC-IGS fragment can be used to identify subgenus level taxonomic units of the larger cyanobacterial community (Miller and McMahon, 2011). The *MspI* fragments were sized using denaturing capillary electrophoresis [ABI 37306l DNA Analyzer; University of Wisconsin Biotechnology Center (UWBC)]. For each sample, triplicate electropherogram profiles were analyzed using GeneMarker® (SoftGenetics) software v 1.5. In addition, a script developed in the R Statistics Environment was used to distinguish potential peaks from baseline noise (Jones and McMahon, 2009; Jones et al., 2012). Relative abundance data from this script was calculated by dividing the height of each peak by the sum of peak heights per sample. Aligned, overlapping peaks were binned into subgenus taxonomic units (Miller and McMahon, 2011). Fragment lengths were matched to an *in silico* digested database of PC-IGS sequences using the Phylogenetic Assignment Tool (https://secure.limnology.wisc.edu/trflp/) (Kent et al., 2003). These taxa were then named based on the genus and base pair length of the PC-IGS fragment identified. For example, “Mic215” represents a *Microcystis* genotype identified by a PC-IGS fragment length of 215 base pairs.

### Lake physics calculations

Lake physics characteristics were calculated using MatLab v2012a (2012)^1^ and the previously described Lake Analyzer program (Read et al., 2011). Briefly, Wedderburn Number (W), Schmidt Stability (SS), Lake Number (L_N_), and mixed layer depth (Z_mix_) were estimated from water temperature profiles and meteorological data collected at the time of sampling, as well as lake bathymetry. SS represents the resistance of lake mixing due to the energy stored within the lake (i.e., how stratified the lake is), W describes the likelihood of upwelling under stratified conditions, and L_N_ represents the amount of wind induced mixing within the lake. Both W and L_N_ are affected by the water friction velocity (u^*^) that is due to wind forcing. Lake Analyzer also calculates the buoyancy frequency (N^2^), which describes the water column stability based on density gradients. Very simply, these indices are expected to increase as a lake becomes more thermally stratified and takes more energy and wind forcing to cause the water column to turnover.

### Statistical methods

The NTL-LTER program collects integrated 8 m samples from the surface waters for phytoplankton counts, but chemical data are collected at discrete depths ranging from 0 m to the bottom of the lake (and include 0, 2, 4, and 8 m). Therefore, in this analysis, we integrated all chemical data between 0 and 8 m for comparison to the phytoplankton communities. In addition, all temperature, dissolved oxygen, and pH measurements were collected at 1 m intervals between 0 and 8 m and were integrated for statistical analyses as well. All LTER-MO samples that were collected for cyanobacterial community composition were collected between 0 and 12 m, so direct comparisons between the two LTER datasets (0–8 m) are confounded by this discrepancy.

For temporal comparisons, we used the Kruskal–Wallis (K–W) non-parametric test instead of analysis of variance (ANOVA) to determine if seasonal means were significantly different (*p* < 0.05) since not all data were normally distributed. All K–W comparisons were performed in MatLab v2012a using the *kruskalwallis* function. All regression models were performed using the *LinearModel.fit* function in MatLab v2-12a.

## Results

### Defining the toxic phase

**Figure 1:**
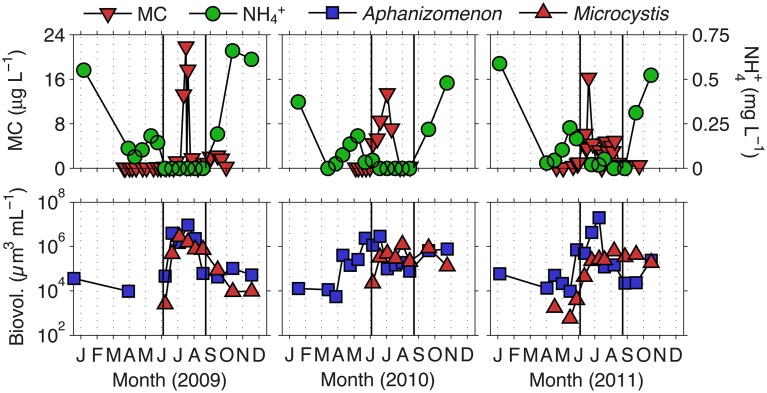
**Ammonium and total microcystin concentrations, as well as *Aphanizomenon* and *Microcystis* biovolume, in Lake Mendota for the years 2009–2011**. Solid lines indicate the beginning (day 170) and end (day 250) of what we have defined as the toxic phase.

**Figure 2:**
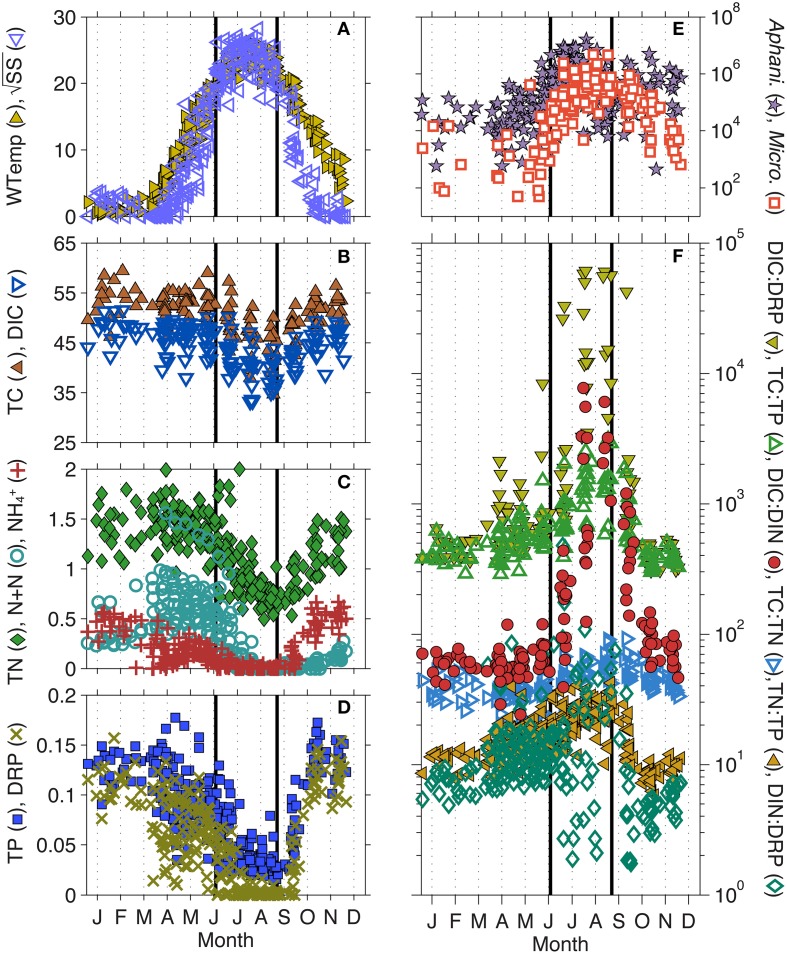
**Intra-annual changes in (A) water temperature (WTemp) and Schmidt Stability (SS), (B) total carbon (TC) and dissolved inorganic C (DIC), (C) total nitrogen (TN), nitrate + nitrite (N + N), and ammonium (NH^+^_4_), (D) total phosphorus (TP) and dissolved reactive P (DRP), (E) Microcystis and Aphanizomenon biovolume, (F) ratios of TC: TN, TC: TP, TP: TN, as well as DIC: DRP, DIC: dissolved inorganic N (DIN), and DIN: DRP**. All plots represent data collected between 1995–2010 by the North Temperate Lakes-Long Term Ecological Research (NTL-LTER) program. Solid lines indicate the beginning (day 170) and end (day 250) of what we have defined as the toxic phase. See Table 1 for units.

We previously defined the toxic phase in Lake Mendota as the time period in which MC concentrations were above 1 μg L^−1^, the World Health Organization's (WHO) level for safe drinking water (Who, 1999; Beversdorf et al., 2013). Deep Hole measurements of MC were conducted during 2009–2011 between the months of April and October. Based on the 3 years of MC data observed, we defined the toxic phase of Lake Mendota to occur between days 170 and 250 (June 19th to September 7th) where mean MC concentrations were significantly above 1 μg L^−1^ ± standard error (Figure 1). Additionally, days 1–169 were termed the pre-toxic phase and days 251–365 were termed the post-toxic phase. In these years, we noted that *Microcystis* biovolume and MC concentrations were most prevalent when NH^+^_4_ was not detectable in Lake Mendota. *Aphanizomenon* emerged as the most abundant cyanobacteria in every year during the decline of NH^+^_4_, though N + N concentrations were still relatively high, suggesting that the rapid decline in NH^+^_4_ actually induced the lake to undergo temporary N limitation. This phenomenon occurred throughout the entire LTER sampling period as well (1995–2010) (Figure 2) and is likely associated with the ability to fix N_2_, which was measured in 2010 and 2011 (Beversdorf et al., 2013). Because we only had MC measurements for 3 years, we used LTER samples (collected at biweekly time-scales from 1995 to 2010), to interrogate these phases further [pre-toxic phase (*n* = 122); toxic phase (*n* = 100); post-toxic phase (*n* = 55)].

### Pre-toxic to toxic phase transition

When parameter means were compared between the three lake phases described, most were significantly different, likely due to the strong seasonal gradients found in Lake Mendota (Table 1, standard deviations and ranges in Table S1). The most significant changes occurred during the transition from the pre-toxic to toxic phase, as this is essentially the seasonal change from winter thaw to spring to summer stratification in the lake. As expected, physical lake parameters such as L_N_, W, SS, and N^2^ all significantly increased as water temperature increased and the lake began to stratify (Figure 2A). As the thermocline developed, the mixed layer and photic zone (i.e., Secchi) depths decreased. Total cyanobacterial biovolume, and all of the major genera except *Synechocystis*, significantly increased as well. Most notably, *Aphanizomenon* was the most abundant genera observed, followed by *Microcystis* (Figure 2E). Along with the increase in cyanobacterial volume, most nutrients significantly decreased (Figures 2B–D). Only DOC and the DIN: DRP ratio did not significantly change during this time, though the mean DIN: DRP ratio increased from 14 to 30 (by mass). The increase in DIN: DRP was associated with precipitous decreases in DRP, while N + N concentrations remained relatively high moving into the toxic phase. TN: TP ratios also significantly increased from 15 to 23, and while DIC only slightly decreased from 46.4 to 40.1 mg L^−1^, the DIC: DIN ratio increased from 61 to 1331 (Figure 2F).

**Table 1:** **Mean values for most abundant Cyanophyta genera, as well as several biological, chemical, and physical parameters measured from the Lake Mendota Deep Hole location between the years 1995–2010**.

	**Pre-toxic**	**Toxic**	**Post-toxic**	**K-W**
**CYANOPHYTA**				
Total biovolume (μm^3^ mL^−1^)	110,000	26,00,000	920,000	a, b, c
*Aphanizomenon* (μm^3^ mL^−1^)	85,000	17,00,000	560,000	a, b, c
*Microcystis* (μm^3^ mL^−1^)	4600	440,000	150,000	a, b, c
*Oscillatoria* (μm^3^ mL^−1^)	1100	110,000	68,000	a, b, c
*Aphanothece* (μm^3^ mL^−1^)	3900	100,000	17,000	a, b, c
*Synechococcus* (μm^3^ mL^−1^)	1900	4400	3500	a, c
*Synechocystis* (μm^3^ mL^−1^)	1600	1200	1700	
**NUTRIENTS**				
TC (mg L^−1^)	52.2	45.9	50.3	a, b, c
TN (μg L^−1^)	1470	980	1060	a, b, c
TP (μg L^−1^)	110	50	110	a, b
TC:TN (by mass)	37	51	49	a, c
TC:TP (by mass)	515	1230	543	a, b
TN:TP (by mass)	15	23	12	a, b, c
DOC (mg L^−1^)	5.9	6.1	5.8	
DIC (mg L^−1^)	46.4	40.1	44.2	a, b, c
N + N (μg L^−1^)	600	160	60	a, b, c
NH^+^_4_ (μg L^−1^)	210	20	340	a, b, c
DRP (μg L^−1^)	80	10	80	a, b
DIC:DIN (by mass)	61	1331	222	a, b, c
DIC:DRP (by mass)	808	17260	1811	a, b, c
DIN:DRP (by mass)	14	30	7	b, c
DRSi (μg L^−1^)	1190	1560	2000	a, b, c
**PHYSICAL**				
Water temp (°C)	8.8	23.1	12.9	a, b, c
Dissolved oxygen (mg L^−1^)	12.0	8.6	9.0	a, c
pH (–log [H^+^])	8.4	8.9	8.4	a, b
Secchi (m)	5.2	2.4	3.3	a, b, c
Lake number (unitless)	0.57	3.08	0.38	a, b
Wedderburn number (unitless)	1.23	5.54	1.80	a, b
Schmidt stability (J m^−2^)	66	537	72	a, b
u^*^ (m s^−1^)	0.015	0.015	0.016	
Boyancy frequency (N^2^, s^−2^)	0.001	0.005	0.002	a, b
Mixed layer depth (Z_mix_, m)	12.3	7.5	16.3	a, b, c

*All of the data were split into three time periods (phases) based on the 2009–2011 microcystin data. The toxic phase represents the period when mean microcystin concentrations were significantly greater than 1 μg L^−1^ (days 170–250). Significance between the phases was tested using a Kruskal–Wallis test (K–W; p < 0.05). a, significant difference between pre-toxic and toxic phases; b, significant difference between toxic and post-toxic phases; c, ignificant difference between pre-toxic and post-toxic phases*.

### Differences between pre-toxic and post-toxic phases

We expected fewer parameters to be significantly different between the pre- and post-toxic phases, since spring and fall mixing often lead to seasonal commonalities such as higher nutrients, cooler waters, and a homogenous, well-mixed water column. For example, TP, DRP, TC: TP, pH, and lake physics characteristics related to internal lake mixing (i.e., W, SS, N^2^, Z_mix_, and L_N_) were not significantly different between the pre- and post-toxic phases (Table 1). Conversely, with the exception of *Synechocystis*, all major cyanobacterial genera were significantly higher in the post-toxic phase compared to the pre-toxic phase, including *Microcystis* (Figure 2E). Temperature was also significantly higher in the post-toxic phase by an average of 4.1°C. Of particular interest here, N + N and NH^+^_4_ reversed roles in the pre- and post-toxic phases; N + N concentrations were at their lowest, whereas NH^+^_4_ concentrations were at their highest, during the post-toxic phase (Figure 2C). As a result, the relatively high DRP, and low N + N, concentrations led to the lowest TN: TP and DIN: DRP ratios during the post-toxic phase (~12 and 7, respectively) (Figure 2F).

### Toxic to post-toxic transition

As with the pre-toxic to toxic transition, we expected most physical parameters to significantly change as water temperatures cooled into the post-toxic phase, thermal stratification broke down, and the lake mixed. As such, W, buoyancy frequency (N^2^), L_N_, and SS all significantly decreased (Table 1). Additionally, the average mixed layer depth increased from 7.5 to 16.3 m. With the lake turning over, nutrients also significantly increased as nutrient rich hypolimnetic water became entrained into the epilimnion (Figures 2B–D). As described above, one exception was that N + N remained low (60 μg L^−1^). However, NH^+^_4_ significantly increased from 20 to 340 μg L^−1^. TN: TP decreased from 23 to 12, DIN: DRP decreased from 30 to 7 and DIC: DIN decreased from 1331 to 222. The major cyanobacterial genera also significantly decreased (Table 1, Figure 2E), though *Aphanizomenon* and *Microcystis* remained relatively high well into the post-toxic phase representing approximately 49 and 29%, respectively, of the total cyanobacterial biovolume and cyanobacteria were still the dominant phytoplankton species. Additionally, while the water temperature decreased from the toxic to post-toxic phase, average temperatures remained above 15°C until mid-October (Figure 2A).

### Changes in *Microcystis* genotypes throughout the summer and long term

**Figure 3:**
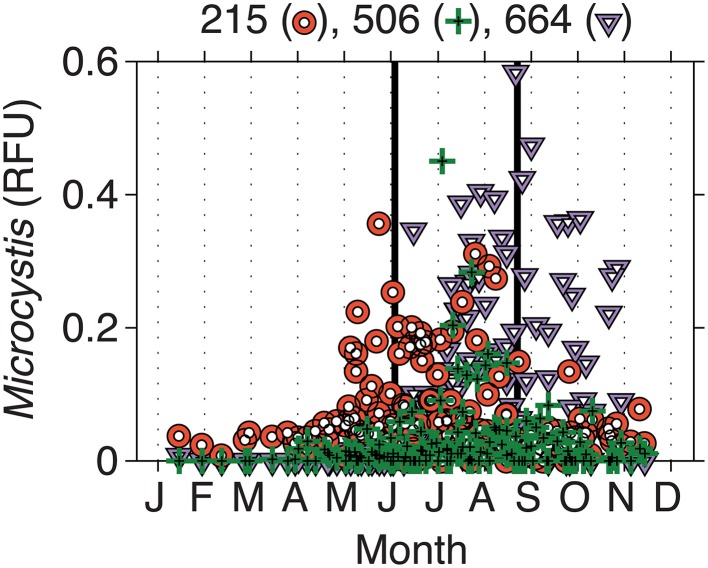
**Intra-annual changes in *Microcystis* genotypes in relative fluorescence units (RFU)**. Biweekly samples were collected by the LTER-Microbial Observatory from 2000 to 2010. Three different genotypes were identified—Mic215, Mic506, and Mic660—and emerged at different times of the year. Solid lines indicate the beginning (day 170) and end (day 250) of what we have defined as the toxic phase.

From 2000 to 2010, the LTER-MO has collected DNA from the Deep Hole location of Lake Mendota. Using the phycocyanin intergenic spacer region (PC-IGS) and comparing the fragments observed to an *in silico* digest, we have identified several abundant taxa at the molecular level. Of interest here, three *Microcystis* taxa (Mic215, Mic506, and Mic660) are of the most abundant cyanobacteria in Lake Mendota, representing, on average, up to ~60% of the cyanobacterial community at times (Figure 3). However, all three taxa (on average) occurred at different times of the year with Mic215 occurring first, followed by Mic506 and then Mic660 (Figure 3). Fitting the data using a Gaussian distribution, the 95% confidence intervals place the peak abundances for Mic215, Mic506, and Mic660 between days 160–200, 210–230, and 240–260, respectively. Because the data are relative to the total cyanobacterial community, it is impossible to determine the absolute abundance of each *Microcystis* genotype moving from the toxic to post-toxic phase. However, on average, the sum of the *Microcystis* genotypes made up 20% of the cyanobacterial community during the toxic and post-toxic phases (Table 2, standard deviations and ranges in Table S2).

**Table 2:** **Mean values for known *Microcystis* genotypes measured from the Lake Mendota Deep Hole location between the years 2000–2010**.

**Genotype**	**Pre-toxic**	**Toxic**	**Post-toxic**	**K-W**
Microcystis 215 (%)	8.1	5.6	3.7	c
Microcystis 506 (%)	0.5	1.4	1.7	a
Microcystis 660 (%)	0.2	13.0	14.5	a, c
Total Microcystis (%)	8.8	20.0	19.9	a, c

*All of the data were split into three time periods (phases) based on the 2009–2011 microcystin data. The toxic phase represents the period when mean microcystin concentrations were significantly greater than 1 μg L^−1^ (days 170–250). Significance between the phases was tested using a Kruskal–Wallis test (K–W; p < 0.05). a, significant difference between pre-toxic and toxic phases; c, significant difference between pre-toxic and post-toxic phases*.

**Figure 4:**
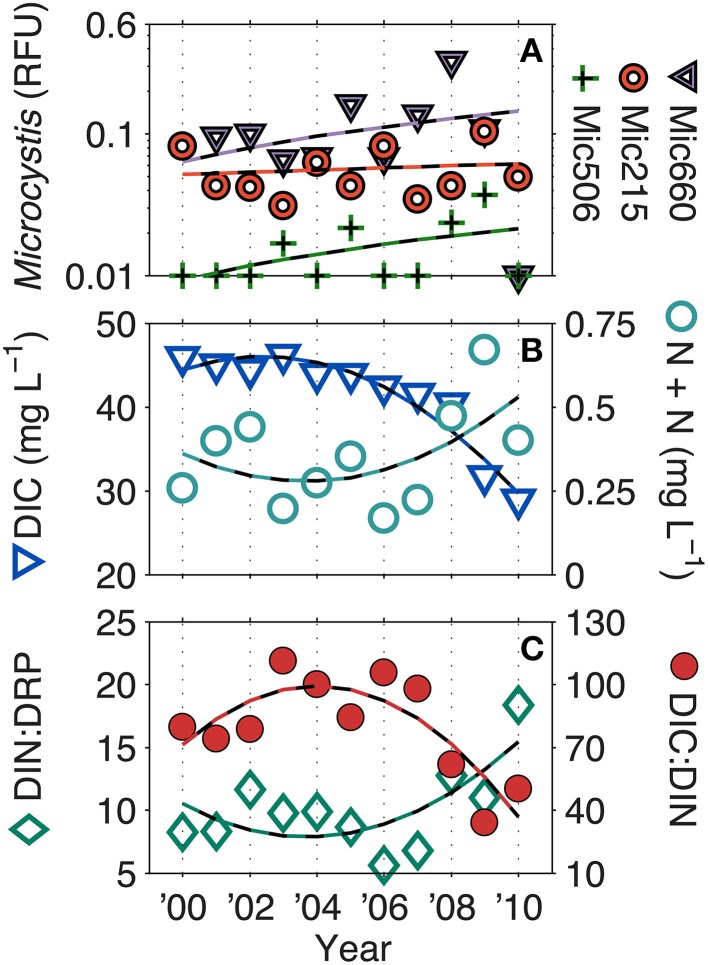
**Long-term trends in (A) *Microcystis* genotypes, (B) dissolved inorganic carbon (DIC), nitrate + nitrite (N + N), and the (C) DIC: DIN and DIC: dissolved reactive phosphorus ratios (DRP) (ratios by mass)**. Dashed lines represent significant (*p* < 0.05) linear correlations for *Microcystis* genotypes and significant second order polynomial regressions for DIC, DIC: DIN, and DIN: DRP. N + N was not significant. Additionally, in 2008, South Central Wisconsin experienced a 100-year flood, which may account for some of biogeochemical changes observed at that time.

Over the LTER time-series (NTL and MO), few parameters have significantly changed. However, all *Microcystis* genotypes have significantly increased (linear, *p* < 0.05), albeit at very low *R*^2^-values (Mic215 = 0.04, Mic506 = 0.12, Mic660 = 0.04). Additionally, DIC (*R*^2^ = 0.93), DIC: DIN (*R*^2^ = 0.68), and DIN: DRP (*R*^2^ = 0.50) have also significantly increased (second order polynomial, *p* < 0.05) (Figure 4). While N + N has not significantly increased (*R*^2^ = 0.30, *p* > 0.05), it has drastically altered the C: N and N: P ratios since 2008, which was also a year of a 100-year flood in South Central Wisconsin (Fitzpatrick et al., 2008).

## Discussion

Using 3 years of observed microcystin measurements, we described three phases in Lake Mendota as pre-toxic, toxic, and post-toxic phases. We chose to use these time frames, rather than the conventional seasons (e.g., spring, summer, autumn), to accurately describe the environmental conditions present before, during, and after toxic cyanobacterial blooms. We then used two long-term datasets to investigate the intra-annual variability in biological, chemical, and physical factors that these phases consistently undergo each year. TN: TP, N + N, NH^+^_4_, DIC: DIN, water temperature, and Secchi depth, among others, were all significantly different in all three phases described. Large external and internal nutrient loading following ice-off in the spring resulted in high DRP, NH^+^_4_, and N + N concentrations, with TN: TP ratios of ~15, during the pre-toxic phase. DIC was also highest in the pre-toxic phase since gases are more soluble in cold water. As Lake Mendota began to stratify moving into the toxic phase, NH^+^_4_ was rapidly taken up, resulting in N stress and N_2_ fixation (Beversdorf et al., 2013). Then, DRP and N + N were converted to biomass and TN: TP ratios were highest during the toxic phase (~23). DIC decreased slightly as water temperatures rose, but the absence of N resulted in extremely high DIC: DIN ratios (>1000 by mass). In the post-toxic phase, DRP and N increased due to lake mixing, but the majority of N was in the form of NH^+^_4_, likely due to summer ammonification in the hypolimnion. DRP concentrations were relatively high compared to that of NH^+^_4_ resulting in very low DIN: DRP and TN: TP ratios (<7 and <12, respectively). Water temperatures decreased resulting in an increase in DIC. However, while decreased in the post-toxic phase, water temperatures were still significantly warmer (by 4.1°C) than in the pre-toxic phase. Thus, *Microcystis*, though decreased overall moving into the post-toxic phase, was still able to account for a major portion of the cyanobacterial community (29% by biovolume, 20% by RFU). Additionally, a different *Microcystis* taxa (Mic660) was dominant during this time period. This might suggest that 1) either Mic660 represents a non-toxic strain of *Microcystis* and/or 2) some environmental factor in the post-toxic phase triggered toxin production to shut down. Herein, we discuss environmental factors that may select for toxic strains of *Microcystis* in lakes or that may trigger MC synthesis.

A recent publication by Scott et al. (2013) regarding the occurrence of MC in lakes with low N to P (N: P) ratios (Orihel et al., 2012) discussed the interpretation of total N (TN) and total P (TP) ratios in eutrophic, toxic lakes. Their analysis suggested that moderate TN: TP ratios (12–23 by mass) were a better predictor of microcystin levels in lakes and concluded the TN: TP ratios are a reflection of total phytoplankton biomass and not causally linked to MC concentrations. Our results support their premise as TN: TP ratios averaged ~20 during the toxic phase when cyanobacterial biovolume was at its peak. However, those were also the highest TN: TP ratios observed throughout the year (Figure 2). Thus, in Lake Mendota, low TN: TP ratios correlate better to other phytoplankton divisions such as Bacillariophyta, Chlorophyta, Chrysophyta, and Cryptophyta and the percent of Cyanophyta that makes up the whole community increases with increasing TN: TP (Figure S1). Scott et al. (2013) also stated that TN: TP ratios may not be a good indicator of nutrient availability. Again, our results support that premise. In both the pre- and post-toxic phases, TN: TP ratios were relatively low (≤ 15) due to the very high concentrations of P (mostly DRP). In the pre-toxic phase, N + N was three times higher than NH^+^_4_, but NH^+^_4_ was an order of magnitude higher than N + N in the post-toxic phase. During the toxic phase, NH^+^_4_ was the first nutrient to drop below detection. Conversely, DIC remained extremely high in Lake Mendota year round. Thus, all three phases had very different inorganic nutrient regimes, most notably with respect to N speciation, and it appeared that NH^+^_4_ concentrations had a larger impact on N limitation and community changes than N + N (Beversdorf et al., 2013).

**Figure 5:**
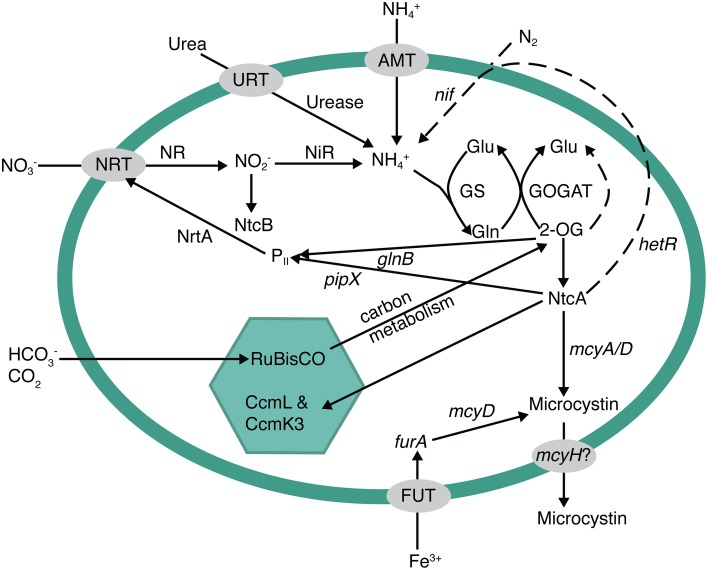
**Metabolic pathways potentially involved in the regulation of microcystin biosynthesis**. Nitrogen metabolism and acquisition, photosynthesis/carbon regulation, and iron uptake have been previously implicated as pathways involved in microcystin expression. NtcA is a major global regulator that links them together. If there is an excess of carbon (either from nitrogen starvation or replete carbon fixation), the buildup of 2-oxoglutarate triggers *ntcA* to turn on nitrogen uptake systems, such as nitrate uptake (*nrtA*) or nitrogen fixation (*hetR*, dotted line pathway), and inhibit carbon fixation. Additionally, NtcA has been shown to bind to the microcystin operon at an *mcyA/D* transcriptional start. Other potential start sites exist in the operon for both iron and light regulation.

Previous research suggested that MC biosynthesis in *Microcystis* is regulated by cellular C and N metabolism (Ginn et al., 2010; Alexova et al., 2011b; Kuniyoshi et al., 2011). Ginn et al. (2010) and Kuniyoshi et al. (2011) suggested that under N stress, the global N regulator, NtcA, may bind to the *mcyA/D* promoter to activate and enhance MC production. Additionally, Downing et al. (2005) reported that microcystin production was regulated by N uptake rates, rather than overall growth rate. Thus, these studies may be causally linked. Under N stressed conditions, NtcA would trigger nitrate uptake and assimilation, which in turn would be reflected in an increase in MC production due to NtcA as well (Figure 5). Transcription of *ntcA*, the global N regulating gene in cyanobacteria, is actually regulated by levels of 2-oxoglutarate (2-OG) within the cell (Muro-Pastor et al., 2001, 2005; Tanigawa et al., 2002). NH^+^_4_ and 2-OG are tightly regulated and both are incorporated into the glutamine synthetase (GS) and glutamate synthase (GOGAT) pathways as the N and C building blocks of amino acids and other C-N molecules (Figure 5). Assimilation of N in the cell must be in the reduced form of NH^+^_4_. Therefore, nitrate, urea, N_2_fixation, etc., must be converted to NH^+^_4_ before entering into the GS-GOGAT pathway where it is eventually assimilated with 2-OG into glutamate and glutamine. Thus, the presence of available N does not necessarily mean that genes controlled by cellular N concentration will be turned off, or down-regulated within a cell. If levels of 2-OG are high, NtcA will activate genes involved in assimilation of other N forms. In addition, if NH^+^_4_ in particular is rapidly drawn down, a lag period may occur before enough N (e.g., nitrate or N_2_) can be reduced to offset 2-OG levels. Thus, N_2_ fixation can occur in the presence of nitrate (suggesting rapid N limitation), as we have seen in this dataset and elsewhere (Flores and Herrero, 2004), further suggesting that the absence of NH^+^_4_ alone can cause N stress. The absence of NH^+^_4_ could be a trigger for toxin production if *ntcA* and *mcy* are coupled as suggested by Ginn et al. (2010) and Kuniyoshi et al. (2011). This could also explain why MC concentrations decrease in the post-toxic phase, even though water temperatures and *Microcystis* biovolume remain relatively high, because epilimnetic NH^+^_4_ rapidly increases with the onset of lake mixing.

In addition to 2-OG regulation of *ntcA* and MC, C metabolism has been implicated in MC production. While most studies have related light levels to growth, or possibly oxidative stress, two of the first studies on MC biosynthesis showed that there are two light-dependent transcription start sites within the *mcyA/D* region of the *mcy* operon and that one region may be constitutively expressed under low light, while the other is up-regulated under high light (Kaebernick et al., 2000, 2002). This would also relate to C and N balance in the cell if, for instance, high light intensity led to increased photosynthesis, C fixation, and C intermediates such as 2-OG, which could then trigger an increase in *ntcA* and *mcy* expression. Additionally, Alexova et al. (2011b) observed that proteins involved in N and C metabolism were differentially expressed in toxic vs. non-toxic strains of *Microcystis aeruginosa*. In particular, toxic strains exhibited an increase in N assimilation proteins and proteins involved in carbon concentration within the carboxysome (CcmL and CcmK3). Thus, toxic strains exhibited differential expression that may be directly related to C/N homeostasis.

Lindell et al. (2002) and Lindell and Post (2001) looked at the expression of *ntcA* under varying NH^+^_4_ concentrations and C: N ratios under conditions relevant to the environment. They found that when NH^+^_4_ concentrations dropped below 1 μM (14 μg L^−1^), *ntcA* was immediately up-regulated. Additionally, small changes in intracellular C: N ratios (e.g., from 6 to 8) resulted in rapid increases in *ntcA* expression. The average NH^+^_4_ concentration in Lake Mendota during the toxic phase was 20 μg L^−1^, which is very close to the 14 μg L^−1^ reported by Lindell and Post (2001). However, given that those experiments were performed with *Prochlorococcus* (a tiny marine picocyanobacteria), it is possible that *Microcystis* has a much larger intracellular NH^+^_4_ demand. Both TC: TN and DIC: DIN ratios were orders of magnitude above the levels reported by Lindell et al. (2002). While TC: TN increased significantly from 37 to 51 from the pre-toxic to toxic phase, it did change into the post-toxic phase. Conversely, the average DIC: DIN ratio increased from 61 to 1331 from the pre-toxic to toxic phase and then dropped back down to 222 in the post-toxic phase. Thus, as with the discussion regarding TN: TP vs. inorganic N and P concentrations, the DIC: DIN ratio is likely to be much more representative of the nutrient availability than TC: TN and suggests that N might be highly limited compared to C, which may again be a trigger for toxin production.

While the underlying cause of MC production remains elusive, our previous studies and data presented here suggest that N and C metabolism could play a major role in MC production in the environment. We have postulated the role of C and N metabolism in toxin production (Figure 5) and suggest that more studies are needed to connect the environmental factors that ultimately control toxin production at the molecular level. An underlying question is, “Why would cyanobacteria produce a C and N rich compound under times of N stress?” In plants, it is well known that altered C: N results in an increase in secondary metabolite synthesis. Since cyanotoxins are secondary metabolites, this is consistent. Under N deficiency, plants tend to build starches and carbohydrates and redirect primary metabolism (Hermans et al., 2006). Alexova et al. (2011b) showed that toxic strains of *Microcystis* did up-regulate the expression of proteins involved in concentrating C in the cyanobacterial carboxysome. Thus, part of the MC response may actually be to redirect primary metabolism to storage molecules for C and possibly N as well. Meissner et al. (2015) also observed an increase in glycogen storage in MC-producing *Microcystis* vs. non-toxic strains under high-light conditions. Interestingly, Meissner et al. also observed a decrease in cellular MC concentrations under those same conditions. One explanation may be that MC binds to proteins in the cell as a result to cellular stress (Zilliges et al., 2011) or as an aid to photosynthesis-related machinery (Makower et al., 2015), and the apparent decrease in the cell is caused by strong covalent binding. This is particularly interesting for field-based studies, as it could account for some of the temporal variability observed in toxic lakes, and calls for new technologies to definitively measure bound MC. Regardless, MC production as a stress response to ROS has also been associated with iron deficiency (Alexova et al., 2011a), which may be part of a larger global response involving iron, N and C metabolism. MC has also been implicated as an allelopathic compound (Holland and Kinnear, 2013) in that, while it costs energy to produce, it gives toxic *Microcystis* a competitive advantage over other phytoplankton in their environment. This may also be represented in our data as three separate genotypes (Mic215, Mic506, and Mic660) dominate the *Microcystis* population at three different times throughout the year (Figure 3) with very different prevailing biological, chemical, and physical characteristics (Figure 2) including high-light conditions (and N stress) occurring during the toxic phase.

Earlier, we alluded to the need for a systematic approach using both field-based observations and transcriptional culture-based studies to ascertain the physio-ecological role for microcystin production. We have shown how C and N metabolism could be connected to MC production at the **ecosystem** level and proposed a **cellular** model for MC regulation/synthesis based on available laboratory studies. Very recent transcriptomic studies have validated, to some extent, these connections, and we suggest that further studies—including mechanistic studies and models, experiments focusing on functional genes, and ecosystem level transcription/-omics—need to focus on the links between C and N metabolism and a potential role of MC as an aid to photosynthesis and ROS protection. Specifically, several studies have shown that MC may be localized within the cell, and may form specific bonds with photosynthetic machinery including the thylakoid membrane. While this provides strong evidence for current hypotheses that suggest MC is involved is ROS protection, C storage, or even as an electron sink, further data are needed to employ reasonable management decisions regarding freshwater resources.

### Conflict of interest statement

The authors declare that the research was conducted in the absence of any commercial or financial relationships that could be construed as a potential conflict of interest.

## Footnotes

^1^(2012). *MATLAB*.Natick, MA: The MathWorks Inc.

## References

[B1] Alexova R. Fujii M. Birch D. Cheng J. Waite T. D. Ferrari B. C.. (2011a). Iron uptake and toxin synthesis in the bloom-forming *Microcystis aeruginosa* under iron limitation. Environ. Microbiol. 13, 1064–1077. 10.1111/j.1462-2920.2010.02412.x 21251177

[B2] Alexova R. Haynes P. A. Ferrari B. C. Neilan B. A. (2011b). Comparative protein expression in different strains of the bloom-forming cyanobacterium *Microcystis aeruginosa*. Mol. Cell. Proteomics 10, 1064–1077 10.1074/mcp.M110.003749 PMC3186190 21610102

[B3] Balbus J. M. Boxall A. B. A. Fenske R. A. McKone T. E. Zeise L. (2013). Implications of global climate change for the assessment and management of human health risks of chemicals in the natural environment. Environ. Toxicol. Chem. 32, 62–78. 10.1002/etc.2046 23147420 PMC3601433

[B4] Beversdorf L. J. Chaston S. D. Miller T. R. McMahon K. D. (2015). Microcystin *mcyA* and *mcyE* gene abundances are not appropriate indicators of microcystin concentrations in lakes. PLoS ONE. 10:e0125353. 10.1371/journal.pone.0125353 25945933 PMC4422731

[B5] Beversdorf L. J. Miller T. R. McMahon K. D. (2013). The role of nitrogen fixation in cyanobacterial bloom toxicity in a temperate, eutrophic lake. PLoS ONE 8:e56103. 10.1371/journal.pone.0056103 23405255 PMC3566065

[B6] Brock T. D. (1985). A Eutrophic Lake - Lake Mendota, Wisconsin. New York, NY: Springer-Verlag 10.1007/978-1-4419-8700-6

[B7] Christiansen G. Molitor C. Philmus B. Kurmayer R. (2008). Nontoxic strains of cyanobacteria are the result of major gene deletion events induced by a transposable element. Mol. Biol. Evol. 25, 1695–1704. 10.1093/molbev/msn120 18502770 PMC2464740

[B8] Crumpton W. G. (1987). A simple and reliable method for making permanent mounts of phytoplankton for light and fluorescence microscopy. Limnol. Oceanogr. 32, 1154–1159 10.4319/lo.1987.32.5.1154

[B9] Downing T. G. Meyer C. Gehringer M. M. van de Venter M. (2005). Microcystin content of *Microcystis aeruginosa* is modulated by nitrogen uptake rate relative to specific growth rate or carbon fixation rate. Environ. Toxicol. 20, 257–262. 10.1002/tox.20106 15892070

[B10] Eaglesham G. K. Norris R. L. Shaw G. R. Smith M. J. Chiswell R. K. Davis B. C. (1999). Use of HPLC-MS/MS to monitor cylindrospermopsin, a blue-green algal toxin, for public health purposes. Environ. Toxicol. 14, 151–154.

[B11] Fitzpatrick F. A. Peppler M. C. Walker J. F. Rose R. J. Waschbusch R. J. Kennedy J. L. (2008). Flood of June 2008 in Southern Wisconsin. Reston, VA: U. S. Geological Survey Scientific Report 5235.

[B12] Flores E. Herrero A. (2004). Assimilatory nitrogen metabolism and its regulation, in The Molecular Biology of Cyanobacteria, ed Bryant D. (Netherlands: Springer), 487–517.

[B13] Ginn H. P. Pearson L. A. Neilan B. A. (2010). NtcA from *Microcystis aeruginosa* PCC 7806 is autoregulatory and binds to the microcystin promoter. Appl. Environ. Microbiol. 76, 4362–4368. 10.1128/AEM.01862-09 20453121 PMC2897459

[B14] Gobler C. J. Davis T. W. Coyne K. J. Boyer G. L. (2007). Interactive influences of nutrient loading, zooplankton grazing, and microcystin synthetase gene expression on cyanobacterial bloom dynamics in a eutrophic New York lake. Harmful Algae 6, 119–133 10.1016/j.hal.2006.08.003

[B15] Harada K.-I. Matsuura K. Suzuki M. Oka H. Watanabe M. F. Oishi S.. (1988). Analysis and purification of toxic peptides from cyanobacteria by reversed-phase high-performance liquid chromatography. J. Chromatograp. 448, 275–283. 10.1016/S0021-9673(01)84589-1 3147286

[B16] Hedman C. J. Krick W. R. Karner Perkins D. A. Harrahy E. A. Sonzogni W. C. (2008). New measurements of cyanobacterial toxins in natural waters using high performance liquid chromatography coupled to tandem mass spectrometry. J. Environ. Qual. 37, 1817–1824. 10.2134/jeq2007.0368 18689743

[B17] Hermans C. Hammond J. P. White P. J. Verbruggen N. (2006). How do plants respond to nutrient shortage by biomass allocation? Trends Plant Sci. 11, 610–617. 10.1016/j.tplants.2006.10.007 17092760

[B18] Holland A. Kinnear S. H. W. (2013). Interpreting the possible ecological role(s) of cyanotoxins. Mar. Drugs 11, 2239–2258. 10.3390/md11072239 23807545 PMC3736421

[B19] Hyenstrand P. Blomqvist P. Pettersson A. (1998). Factors determining cyanobacterial success in aquatic systems: a literature review. Archiv Hydrobiol. Special Issues Adv. Limnol. 51, 41–62.

[B20] Jähnichen S. Ihle T. Petzoldt T. Benndorf J. (2007). Impact of inorganic carbon availability on microcystin production by *Microcystis aeruginosa* PCC 7806. Appl. Environ. Microbiol. 73, 6994–7002. 10.1128/AEM.01253-07 17827326 PMC2074933

[B21] Jones S. E. Cadkin T. A. Newton R. J. McMahon K. D. (2012). Spatial and temporal scales of aquatic bacterial beta diversity. Front. Microbiol. 3:318. 10.3389/fmicb.2012.00318 22969757 PMC3431545

[B22] Jones S. E. McMahon K. D. (2009). Species-sorting may explain an apparent minimal effect of immigration on freshwater bacterial community dynamics. Environ. Microbiol. 11, 905–913. 10.1111/j.1462-2920.2008.01814.x 19040451

[B23] Kaebernick M. Dittmann E. Börner T. Neilan B. A. (2002). Multiple alternate transcripts direct the biosynthesis of microcystin, a cyanobacterial nonribosomal peptide. Appl. Environ. Microbiol. 68, 449–455. 10.1128/AEM.68.2.449-455.2002 11823177 PMC126702

[B24] Kaebernick M. Neilan B. A. Börner T. Dittmann E. (2000). Light and the transcriptional response of the microcystin biosynthesis gene cluster. Appl. Environ. Microbiol. 66, 3387–3392. 10.1128/AEM.66.8.3387-3392.2000 10919796 PMC92160

[B25] Kent A. D. Smith D. J. Benson B. J. Triplett E. W. (2003). Web-based phylogenetic assignment tool for analysis of terminal restriction fragment length polymorphism profiles of microbial communities. Appl. Environ. Microbiol. 69, 6768–6776. 10.1128/AEM.69.11.6768-6776.2003 14602639 PMC262325

[B26] Kotak B. G. Lam A. K. Y. Prepas E. E. Hrudey S. E. (2000). Role of chemical and physical variables in regulating microcystin-LR concentration in phytoplankton of eutrophic lakes. Can. J. Fish. Aquat. Sci. 57, 1584–1593 10.1139/f00-091

[B27] Kuniyoshi T. M. Gonzalez A. Lopez-Gomollon S. Valladares A. Bes M. T. Fillat M. F.. (2011). 2-oxoglutarate enhances NtcA binding activity to promoter regions of the microcystin synthesis gene cluster. FEBS Lett. 585, 3921–3926. 10.1016/j.febslet.2011.10.034 22062155

[B28] Kurmayer R. Christiansen G. Chorus I. (2003). The abundance of microcystin-producing genotypes correlates positively with colony size in *Microcystis* sp. and determines its microcystin net production in lake wannsee. Appl. Environ. Microbiol. 69, 787–795. 10.1128/AEM.69.2.787-795.2003 12570996 PMC143648

[B29] Kurmayer R. Christiansen G. Fastner J. Börner T. (2004). Abundance of active and inactive microcystin genotypes in populations of the toxic cyanobacterium *Planktothrix* spp. Environ. Microbiol. 6, 831–841. 10.1111/j.1462-2920.2004.00626.x 15250885 PMC7619634

[B30] Lathrop R. C. (2007). Perspectives on the eutrophication of the Yahara lakes. Lake Reserv. Manag. 23, 345–365 10.1080/07438140709354023

[B31] Lindell D. Erdner D. Marie D. Prášil O. KoblíŽek M. Le Gall F. (2002). Nitrogen stress response of Prochlorococcus strain PCC 9511 (oxyphotobacteria) involves contrasting regulation of ntcA and amt1. J. Phycol. 38, 1113–1124 10.1046/j.1529-8817.2002.01205.x

[B32] Lindell D. Post A. F. (2001). Ecological aspects of *ntcA* gene expression and its use as an indicator of the nitrogen status of marine *Synechococcus* spp. Appl. Environ. Microbiol. 67, 3340–3349. 10.1128/AEM.67.8.3340-3349.2001 11472902 PMC93026

[B33] Makower A. K. Schuurmans J. M. Groth D. Zilliges Y. Matthijs H. C. P. Dittmann E. (2015). Transcriptomics-aided dissection of the intracellular and extracellular roles of microcystin in *Microcystis aeruginosa* PCC 7806. Appl. Environ. Microbiol. 81, 544–554. 10.1128/AEM.02601-14 25381232 PMC4277579

[B34] Meissner S. Steinhauser D. Dittmann E. (2015). Metabolomic analysis indicates a pivotal role of the hepatotoxin microcystin in high light adaptation of *Microcystis*. Environ. Microbiol. 17, 1497–1509. 10.1111/1462-2920.12565 25041118

[B35] Miller T. R. McMahon K. D. (2011). Genetic diversity of cyanobacteria in four eutrophic lakes. FEMS Microbiol. Ecol. 78, 336–348. 10.1111/j.1574-6941.2011.01162.x 21707672

[B36] Muro-Pastor M. I. Reyes J. C. Florencio F. J. (2001). Cyanobacteria perceive nitrogen status by sensing intracellular 2-oxoglutarate levels. J. Biol. Chem. 276, 38320–38328. 11479309 10.1074/jbc.M105297200

[B37] Muro-Pastor M. I. Reyes J. Florencio F. (2005). Ammonium assimilation in cyanobacteria. Photosyn. Res. 83, 135–150. 10.1007/s11120-004-2082-7 16143848

[B38] Neilan B. A. Pearson L. A. Muenchhoff J. Moffitt M. C. Dittmann E. (2012). Environmental conditions that influence toxin biosynthesis in cyanobacteria. Environ. Microbiol. 15, 1239–1253. 10.1111/j.1462-2920.2012.02729.x 22429476

[B39] Oh H. M. Lee S. J. Kim J. H. Kim H. S. Yoon B. D. (2001). Seasonal variation and indirect monitoring of microcystin concentrations in Daechung Reservoir, Korea. Appl. Environ. Microbiol. 67, 1484–1489. 10.1128/AEM.67.4.1484-1489.2001 11282594 PMC92758

[B40] Ohashi Y. Shi W. Takatani N. Aichi M. Maeda S.-I. Watanabe S.. (2011). Regulation of nitrate assimilation in cyanobacteria. J. Exp. Bot. 62, 1411–1424. 10.1093/jxb/erq427 21282331

[B41] Orihel D. M. Bird D. F. Brylinsky M. Chen H. Donald D. B. Huang D. Y. (2012). High microcystin concentrations occur only at low nitrogen-to-phosphorus ratios in nutrient-rich Canadian lakes. Can. J. Fish. Aquat. Sci. 69, 1457–1462.

[B42] Paerl H. W. Huisman J. (2009). Climate change: a catalyst for global expansion of harmful cyanobacterial blooms. Environ. Microbiol. Rep. 1, 27–37. 10.1111/j.1758-2229.2008.00004.x 23765717

[B43] Paz-Yepes J. Flores E. Herrero A. (2003). Transcriptional effects of the signal transduction protein PII (glnB gene product) on NtcA-dependent genes in *Synechococcus* sp. PCC 7942. FEBS Lett. 543, 42–46. 10.1016/S0014-5793(03)00384-3 12753902

[B44] Rantala A. Rajaniemi-Wacklin P. Lyra C. Lepistö L. Rintala J. Mankiewicz-Boczek J.. (2006). Detection of microcystin-producing cyanobacteria in Finnish Lakes with genus-specific microcystin synthetase gene E (*mcyE*) PCR and associations with environmental factors. Appl. Environ. Microbiol. 72, 6101–6110. 10.1128/AEM.01058-06 16957235 PMC1563646

[B45] Read J. S. Hamilton D. P. Jones I. D. Muraoka K. Winslow L. A. Kroiss R. (2011). Derivation of lake mixing and stratification indices from high-resolution lake buoy data. Environ. Model. Softw. 26, 1325–1336 10.1016/j.envsoft.2011.05.006

[B46] Rinta-Kanto J. M. Konopko E. A. Debruyn J. M. Bourbonniere R. A. Boyer G. L. Wilhelm S. W. (2009). Lake Erie microcystis: relationship between microcystin production, dynamics of genotypes and environmental parameters in a large lake. Harmful Algae 8, 665–673 10.1016/j.hal.2008.12.004

[B47] Scott J. T. McCarthy M. J. Otten T. G. Steffen M. M. Baker B. C. Grantz E. M. (2013). Comment: an alternative interpretation of the relationship between TN:TP and microcystins in Canadian lakes. Can. J. Fish. Aquat. Sci. 70, 1265–1268 10.1139/cjfas-2012-0490

[B48] Sevilla E. Martin-Luna B. Vela L. Teresa Bes M. Luisa Peleato M. Fillat M. (2010). Microcystin-LR synthesis as response to nitrogen: transcriptional analysis of the *mcyD* gene in *Microcystis aeruginosa* PCC7806. Ecotoxicology 19, 1167–1173. 10.1007/s10646-010-0500-5 20532619

[B49] Sipari H. Rantala-Ylinen A. Jokela J. Oksanen I. Sivonen K. (2010). Development of a chip assay and quantitative PCR for detecting microcystin synthetase E gene expression. Appl. Environ. Microbiol. 76, 3797–3805. 10.1128/AEM.00452-10 20400558 PMC2893508

[B50] Tanigawa R. Shirokane M. Maeda S.-I. Omata T. Tanaka K. Takahashi H. (2002). Transcriptional activation of NtcA-dependent promoters of *Synechococcus* sp. PCC 7942 by 2-oxoglutarate *in vitro*. Proc. Natl. Acad. Sci. U.S.A. 99, 4251–4255. 10.1073/pnas.072587199 11917135 PMC123634

[B51] Tillett D. Dittmann E. Erhard M. Von Döhren H. Börner T. Neilan B. A. (2000). Structural organization of microcystin biosynthesis in *Microcystis aeruginosa* PCC7806: an integrated peptide–polyketide synthetase system. Chem. Biol. 7, 753–764. 10.1016/S1074-5521(00)00021-1 11033079

[B52] Vázquez-Bermúdez M. A. F. Herrero A. Flores E. (2002). 2-Oxoglutarate increases the binding affinity of the NtcA (nitrogen control) transcription factor for the *Synechococcus* glnA promoter. FEBS Lett. 512, 71–74. 10.1016/S0014-5793(02)02219-6 11852054

[B53] Who (1999). Toxic Cyanobacteria in Water: A Guide to Their Public Health Consequences, Monitoring and Management. London; New York: E and FN Spon.

[B54] Wicks R. J. Thiel P. G. (1990). Environmental factors affecting the production of peptide toxins in floating scums of the cyanobacterium *Microcystis aeruginosa* in a hypertrophic African reservoir. Environ. Sci. Technol. 24, 1413–1418 10.1021/es00079a017

[B55] Wood S. A. Rueckert A. Hamilton D. P. Cary S. C. Dietrich D. R. (2011). Switching toxin production on and off: intermittent microcystin synthesis in a *Microcystis* bloom. Environ. Microbiol. Rep. 3, 118–124. 10.1111/j.1758-2229.2010.00196.x 23761240

[B56] Yoshida M. Yoshida T. Takashima Y. Hosoda N. Hiroishi S. (2007). Dynamics of microcystin-producing and non-microcystin-producing *Microcystis* populations is correlated with nitrate concentration in a Japanese lake. FEMS Microbiol. Lett. 266, 49–53. 10.1111/j.1574-6968.2006.00496.x 17092296

[B57] Young F. M. Thomson C. Metcalf J. S. Lucocq J. M. Codd G. A. (2005). Immunogold localisation of microcystins in cryosectioned cells of *Microcystis*. J. Struct. Biol. 151, 208–214. 10.1016/j.jsb.2005.05.007 16054393

[B58] Zilliges Y. Kehr J. C. Meissner S. Ishida K. Mikkat S. Hagemann M.. (2011). The cyanobacterial hepatotoxin microcystin binds to proteins and increases the fitness of *Microcystis* under oxidative stress conditions. PLoS ONE 6:e17615. 10.1371/journal.pone.0017615 21445264 PMC3060824

