# Supplementary material for: Long-term monitoring reveals carbon–nitrogen metabolism key to microcystin production in eutrophic lakes
Source: Front Microbiol. 2015 May 12;6:456. doi: 10.3389/fmicb.2015.00456 (PMC4428211; doi:10.3389/fmicb.2015.00456)
Supplement: Supplementary file 1 [file Table1.PDF]

Table S1: Standard deviation and range of Cyanophyta biovolume from the Lake Mendota Deep Hole location between the years 1995-2010. All of the data were split into three time periods (phases) based on the 2009-2011 microcystin data. The toxic phase represents the period when mean microcystin concentrations were significantly greater than 1 µg L-1 (days 170-250). Significance between the phases was tested using a Kruskal-Wallis test (K-W; $p < 0.05$).

**Cyanophyta**	**Pre-toxic**	**Toxic**	**Post-toxic**	**K-W**
Total biovolume	290000	3000000	1000000	a, b, c
	0-2500000	2000-17000000	3600-5000000	
Aphanizomenon	260000	2800000	920000	a, b, c
	0-2300000	0-16000000	0-5000000	
Microcystis	39000	760000	210000	a, b, c
	0-400000	0-4800000	650-890000	
Oscillatoria	4700	540000	380000	a, b, c
	0-44000	0-4300000	0-2600000	
Aphanothece	16000	280000	38000	a, b, c
	0-130000	0-2200000	0-220000	
Synechococcus	2300	5000	3900	a, c
	0-9300	0-25000	0-21000	
Synechocystis	5000	2000	3100	
	0-46000	0-9500	0-11000	

a = significant difference between Pre and Toxic; b = significant difference between Toxic and Post; c = significant difference between Pre and Post phases
