# Supplementary material for: Long-term monitoring reveals carbon–nitrogen metabolism key to microcystin production in eutrophic lakes
Source: Front Microbiol. 2015 May 12;6:456. doi: 10.3389/fmicb.2015.00456 (PMC4428211; doi:10.3389/fmicb.2015.00456)
Supplement: Supplementary file 2 [file Table2.PDF]

Table S2: Standard deviation and range of nutrients concentrations from the Lake Mendota Deep Hole location between the years 1995-2010. All of the data were split into three time periods (phases) based on the 2009-2011 microcystin data. The toxic phase represents the period when mean microcystin concentrations were significantly greater than 1 µg L-1 (days 170-250). Significance between the phases was tested using a Kruskal-Wallis test (K-W; $p < 0.05$).

Nutrients	Pre-toxic	Toxic	Post-toxic	K-W
TC	3.9	5.9	3.2	a, b, c
	32.9-59.4	23.7-57.3	42.5-56.4	
TN	260	330	260	a, b, c
	960-2600	500-2200	530-1500	
TP	30	30	45	a, b
	35-180	15-130	21-170	
TC:TN	6.5	14.4	11.7	a, c
	21.0-54.2	23.7-90.2	33.3-91.2	
TC:TP	180	640	360	a, b
	290-1500	390-3100	300-19000	
TN:TP	5.0	7.1	6.0	a, b, c
	7.8-39.6	10.0-42.2	5.8-30.2	
DOC	0.8	1.2	0.6	
	4.5-9.7	3.6-11.1	4.6-7.1	
DIC	2.9	5.1	3.0	a, b, c
	37.9-51.5	19.8-50.2	38.4-49.4	
NO_3NO_2	260	240	65	a, c
	230-1600	0-1100	0-280	
NH_4^+	140	35	20	a, b, c
	0-570	0-200	0-670	
DRP	30	20	50	a, b
	6-160	0-68	0-150	
DIC:DIN	15.5	2000	280	a, b, c
	24.3-119.3	39.2-7700	46.4-1200	
DIC:DRP	1040	21000	7000	a, b, c
	360-8300	750-61000	310-42000	
DIN:DRP	12.3	66.5	11.5	b, c
	4.9-84.8	0-475	1.7-74.5	
DRSi	1300	670	1300	a, b, c
	0-5200	450-3700	62-5500	

a = significant difference between Pre and Toxic; b = significant difference between Toxic and Post; c = significant difference between Pre and Post phases
