# Supplementary material for: Long-term monitoring reveals carbon–nitrogen metabolism key to microcystin production in eutrophic lakes
Source: Front Microbiol. 2015 May 12;6:456. doi: 10.3389/fmicb.2015.00456 (PMC4428211; doi:10.3389/fmicb.2015.00456)
Supplement: Supplementary file 3 [file Table3.PDF]

Table S3: Standard deviation and range of physical characteristics from the Lake Mendota Deep Hole location between the years 1995-2010. All of the data were split into three time periods (phases) based on the 2009-2011 microcystin data. The toxic phase represents the period when mean microcystin concentrations were significantly greater than 1 μg L-1 (days 170-250). Significance between the phases was tested using a Kruskal-Wallis test (K-W; $p < 0.05$).

Physical	Pre-toxic	Toxic	Post-toxic	K-W
Water temp	5.8	1.6	5.1	a, b, c
	0.4-19.7	19.1-26.3	2.3-21.9	
Dissolved oxygen	2.2	1.3	1.7	a, c
	8.6-17.7	5.5-12.0	5.8-12.2	
pH	0.2	0.2	0.3	a, b
	7.3-9.1	8.4-9.4	7.7-9.0	
Secchi	2.6	1.2	1.3	a, b, c
	0.9-13.4	0.6-7.8	1.3-7.1	
Lake number	3.26	8.42	1.37	a, b
	-0.02-29.6	0.05-65.3	-0.01-9.1	
Wedderburn number	6.60	8.10	5.54	a, b
	-1.2-62.8	0.02-30.2	-1.65-35.0	
Schmidt stability	66	120	110	a, b
	-3.8-370	280-800	-7.7-0.03	
$u*$	0.02	0.01	0.01	
	0.00-0.04	0.00-0.05	0.00-0.03	
Boyancy frequency (N^2)	0.001	0.003	0.002	a, b
	-0.00002-0.008	0.001-0.016	-0.0001-0.012	
Mixed layer depth (Z_{mix})	12.7	3.4	7.9	a, b, c
	0.5-24.0	5.4-22.1	0.5-24.0	

a = significant difference between Pre and Toxic; b = significant difference between Toxic and Post; c = significant difference between Pre and Post phases
