# Supplementary material for: Long-term monitoring reveals carbon–nitrogen metabolism key to microcystin production in eutrophic lakes
Source: Front Microbiol. 2015 May 12;6:456. doi: 10.3389/fmicb.2015.00456 (PMC4428211; doi:10.3389/fmicb.2015.00456)
Supplement: Supplementary file 4 [file Table4.PDF]

**Genotype**	**Pre-toxic**	**Toxic**	**Post-toxic**	**K-W**
Microcystis 215 (%)	8.0	5.4	3.2	c
	(0.9 - 35.8)	(0.0 - 20.2)	(0.0 - 14.9)	
Microcystis 506 (%)	0.7	1.7	2.4	a
	(0.0 - 2.9)	(0.0 - 7.4)	(0.0 - 8.3)	
Microcystis 660 (%)	0.5	14.2	14.1	a, c
	(0.0 - 1.5)	(0.0 - 58.3)	(0.0 - 47.3)	
Total Microcystis (%)	8.2	14.0	14.2	a, c
