# Supplementary figures and images for: Long-term monitoring reveals carbon–nitrogen metabolism key to microcystin production in eutrophic lakes
Source: Front Microbiol. 2015 May 12;6:456. doi: 10.3389/fmicb.2015.00456 (PMC4428211; doi:10.3389/fmicb.2015.00456)

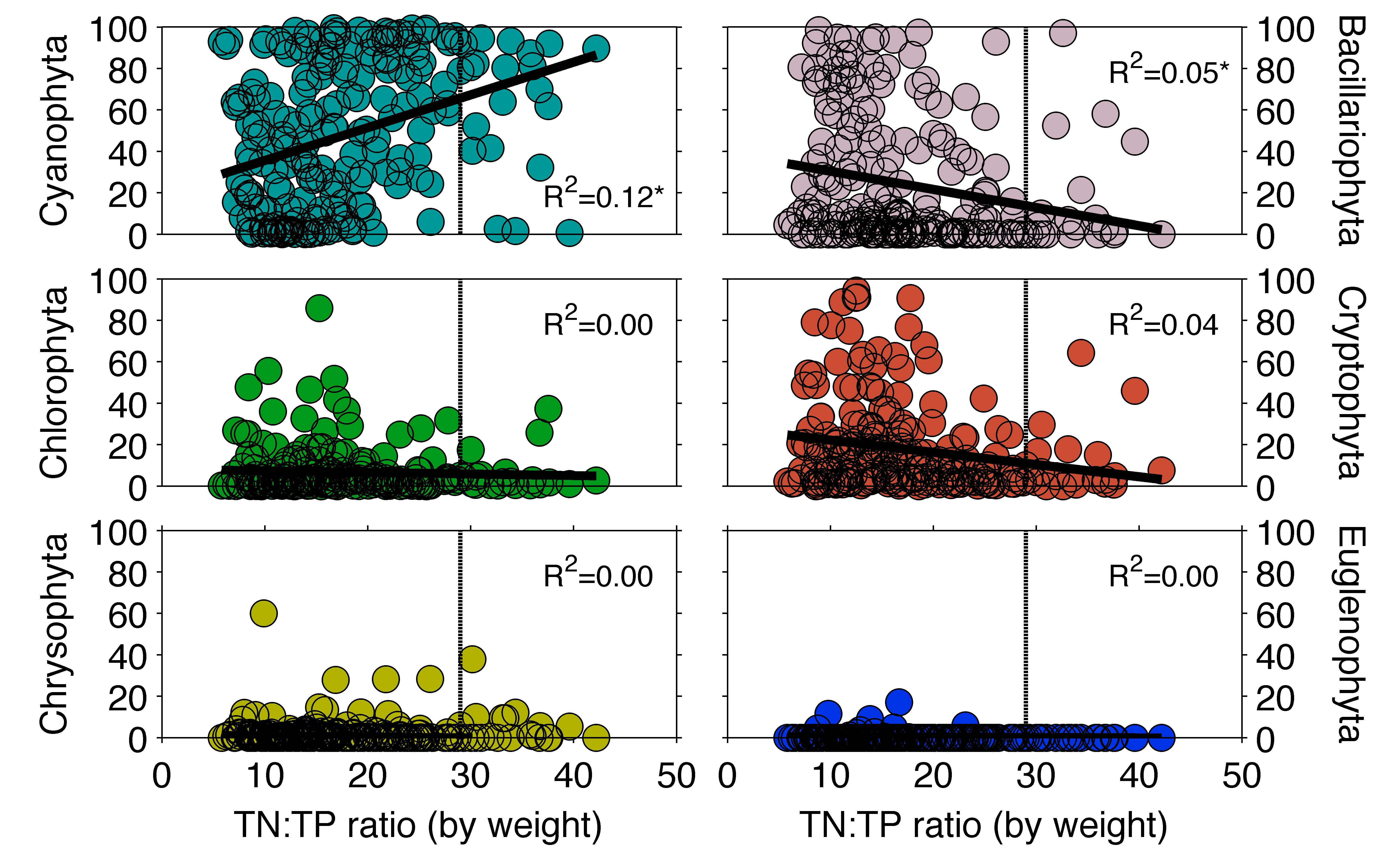

Supplement: Supplementary file 5 [file Image1.TIF]
